# Repercussions of the Maternal Obesogenic Diet on the Oxidative Balance and Pancreatic Metabolism in Male Juvenile Offspring

**DOI:** 10.3390/nu17030578

**Published:** 2025-02-05

**Authors:** Wellington de Almeida Oliveira, Gizele Santiago de Moura Silva, Ramon Nascimento da Silva, José Winglinson Oliveira Santos, Leticia da Silva Pacheco, Deisiane de Araújo Correia, Maria Daniele Teixeira Beltrão de Lemos, Francisco Carlos Amanajás de Aguiar Júnior, Thaynan Raquel dos Prazeres Oliveira, Claudia Jacques Lagranha, Mariana Pinheiro Fernandes

**Affiliations:** 1 Graduate Program of Biochemistry and Physiology, Federal University of Pernambuco, Recife 55670-901, PE, Brazil; wellington.almeidaoliveira@ufpe.br (W.d.A.O.); daniele.lemos@ufpe.br (M.D.T.B.d.L.); claudia.lagranha@ufpe.br (C.J.L.); 2 Graduate Program of Nutrition, Federal University of Pernambuco, Recife 55670-901, PE, Brazil; gizele.santiago@ufpe.br (G.S.d.M.S.); ramon.nsilva@ufpe.br (R.N.d.S.); leticia.pacheco@ufpe.br (L.d.S.P.); 3 Laboratory of Biochemistry and Exercise Biochemistry, Department of Physical Education and Sports Science, Federal University of Pernambuco, Rua Alto do Reservatório, s/n—CEP, Vitória de Santo Antão 55608-680, PE, Brazil; winglinson.oliveira@ufpe.br (J.W.O.S.); deisiane.correia@ufpe.br (D.d.A.C.); 4 Graduate Program Multicenter in Physiological Sciences, Federal University of Pernambuco, Vitória de Santo Antão 55608-680, PE, Brazil; 5 Graduate Program of Nutrition, Physical Activity and Phenotypic Plasticity, Federal University of Pernambuco, Vitória de Santo Antão 55608-680, PE, Brazil; thaynan.raquel@ufpe.br; 6 Professional Master Program in Biology Teaching, Federal University of Pernambuco, Recife 55670-901, PE, Brazil; francisco.amanajas@ufpe.br; 7 Graduate Program of Neuropsychiatry and Behavioral Sciences Graduate Program, Federal University of Pernambuco, Recife 50670-901, PE, Brazil

**Keywords:** mitochondria, obesity, insulin resistance, oxidative balance

## Abstract

**Background/Objectives**: The consumption of diets with high fat, salt, and sugar content has been associated with increasing the risk of developing a range of pathologies, including cardiovascular disease, obesity, and diabetes. Furthermore, there is growing evidence to suggest a relationship between variation in the nutritional environment and pancreatic dysregulation, which may be a consequence of oxidative stress. This study aimed to examine the effects of a high-fat, high-carbohydrate (obesogenic) maternal diet during pregnancy and lactation on the metabolic health and pancreatic structure of rat offspring. **Methods**: Pregnant rats were divided into two groups: one fed a standard diet and the other an obesogenic diet. After weaning, male pups from both groups were fed the same diet until they were 30 days old, which is when they were euthanized. **Results**: Metabolic and murinometric changes: Increased body weight and pancreas size, elevated blood glucose and cholesterol levels, and reduced glucose tolerance (which is indicative of the beginning of insulin resistance). Oxidative stress: Higher levels of oxidative damage markers and decreased antioxidants in the pancreas, suggesting a state of oxidative stress in this organ. Changes in pancreatic structure: Increased size and number of pancreatic islets and decreased size and number of pancreatic acini. **Conclusions**: A maternal obesogenic diet induces metabolic alterations, increases oxidative stress, and causes changes in the structure of the pancreas in rat offspring, suggesting a higher risk of developing metabolic diseases such as type 2 diabetes in adulthood.

## 1. Introduction

The prevalence of obesity is increasing worldwide. According to the World Health Organization (WHO), from 1990 to 2022, the percentage of children and adolescents aged 5–19 years living with obesity increased four-fold from 2% to 8% globally, while the percentage of adults 18 years of age and older living with obesity more than doubled from 7% to 16% [1]. In Brazil, according to the Brazilian Geography and Statistics (IBGE), the prevalence of obesity in adolescents aged 15 to 17 years in Brazil is 19.4% and 6.7%, respectively; in addition, data from the same survey show that the highest prevalence of obese women is in those between 25 and 50 years of age [2].

Obesity is associated with conditions such as dyslipidemia, increased risk of hypertension, and type 2 diabetes. Type 2 diabetes is characterized by a reduction in the ability to absorb glucose due to a reduction in the presence of glucose transporters (GLUTs). The genesis of this pathology may be linked to changes in the pancreas, particularly in the beta cells that secrete insulin [3]. A study carried out by [4], using a model of a diet rich in refined carbohydrates, showed that the animals subjected to this insult showed hypertrophy and the presence of inflammatory cells in the pancreas, as well as an increase in the number and diameter of the islets [4]. Another study carried out by [5] on Sprague Dawley rats showed an increase in the expression of uncoupling protein 2 (UCP2), which is associated with a defense mechanism against increased levels of reactive oxygen species (ROS), suggesting a possible role for ROS in the pathogenesis of the diseases [5]. Additionally, a study conducted by [3] demonstrated that the offspring of obese mothers exhibited elevated levels of UCP2 expression in the pancreas.

Pancreatic dysfunction has been linked to the onset of diabetes and peripheral insulin resistance. Regarding the latter, although this resistance is associated with the expression of insulin receptors in peripheral tissue, some studies have proposed that reactive oxygen species (ROS) and reactive nitrogen species (RNS) may impair insulin signal transduction, thereby compromising insulin homeostasis and glucose metabolism. [6,7]. In an in vitro study with pancreatic beta cells, the addition of mitochondrial substrates to the culture medium revealed that the substrates glutamine, 2-amino-2-norbornanecarboxylic acid (BHC), dimethylglutamate (DMG), and alpha-ketoisocaproic acid (alpha-KIC), when present at a basal concentration of glucose, resulted in an increase in glucose uptake and a subsequent interference with insulin secretion. Furthermore, the same study demonstrated that glucose oxidation is diminished in islets derived from obese animals. In vivo studies have already identified that obese animals exhibit a reduction in UCP2 expression, as well as an increase in lipid peroxidation and protein oxidation and a reduction in antioxidant activity, with a concomitant decrease in superoxide dismutase (SOD) activity and reduced glutathione (GSH) levels. [3,4]. Another study conducted by [8] examined the impact of obesogenic diet consumption on gene expression. The findings revealed an increase in glutathione peroxidase 1 (Gpx1), nuclear factor erythroid 2-related factor 2 (NRF2), peroxisome proliferator activated receptor gamma, and coactivator 1 alpha (Ppargc1a) gene expression, suggesting a potential biological mechanism through which to counteract the surge in reactive species in pancreatic tissue.

Therefore, our hypothesis is that a maternal obesogenic diet causes cellular damage due to oxidative imbalance, which alters pancreatic morphology and the biochemical profile of the juvenile offspring. It is evident that nutritional insults can result in a range of physiological alterations that may impair the quality of life of organisms. However, studies are needed to assess whether changes in the maternal nutritional environment during critical periods of development reflect on the offspring’s metabolism. Thus, the objective of this study was to examine the impact of a maternal obesogenic diet during the pregnancy and lactation periods on the oxidative balance, metabolism, and pancreatic structural architecture in male juvenile offspring.

## 2. Materials and Methods

### 2.1. The Ethical Issues Surrounding the Use of Animals in Research

This project was granted ethical approval by the Ethics Committee for the Use of Animals (CEUA) of the Biosciences Centre of the Federal University of Pernambuco, with approval granted under protocol number 0050/2023. This study employed a total of eight female albino Wistar rats. The rats were not related, were between 90 and 120 days old, weighed between 220 and 250 g, and had not given birth previously. The animals were maintained in an experimental animal facility with a temperature of 23 °C ± 2 °C, a 12/12 light–dark cycle, and unrestricted access to water and food. To ascertain the estrus cycle of the rats, vaginal swabs were employed as a monitoring tool. During the estrus phase, the females were mated at a ratio of two females to one male. To diagnose pregnancy, vaginal swabs were taken, and body weight gain was assessed [9].

### 2.2. Dietary Manipulation and Experimental Groups

During the pregnancy and lactation periods, rats received either a Presence^®^ vivarium diet (n = 4 animals) or an obesogenic diet (n = 4 animals). Two experimental batches were carried out, with four mothers mated per group, mating being carried out in two different moments. The diet was formulated to contain a high fat and carbohydrate content (see Table 1). Additionally, free condensed milk was provided (carbohydrates: 17.7%, proteins: 9.8%, and lipids: 17.7%). Following the 21-day lactation period, only the male offspring were utilized in the subsequent experiments and were fed a Presence^®^ vivarium diet (Table 1). The subjects were also divided into two groups. The control diet group (CD, n = 8 animals) and the obesogenic diet group (OD, n = 8 animals) were euthanized at 30 days of age for subsequent analyses.

### 2.3. Body Weight of the Offspring and Pancreas

The body weight, abdominal circumference, and naso-anal length of the subjects were assessed at the time of euthanasia, and the Lee Index was calculated using the formula ∛ body weight/naso-anal length. An S-Marte digital electronic scale, model S-1000, with a maximum capacity of 1000 g and a sensitivity of 0.01 g, was employed for the assessment of weight. Subsequently, the weight of the pancreas was determined following the collection of the tissue.

### 2.4. Serum Biochemical Profile of Offspring and Oral Glucose Tolerance Test (OGTT)

To assess the concentrations of glucose, total cholesterol, low-density lipoprotein (LDL), high-density lipoprotein (HDL), and triglycerides in the offspring’s blood, colorimetric kits from Labtest^®^ were employed, utilizing serum samples for the measurements and are expressed in mg/dL. To analyze GGT, the animals were fasted for 12 h in accordance with the heat/dark cycle. Subsequently, blood was collected from incisions made at the tip of the animal’s tail. The initial blood sample was obtained at time zero, which corresponds to fasting glucose levels. Subsequently, a 50% glucose solution (Equiplex Pharmaceutical Limited, Goiânia, GO, Brazil) was administered intraperitoneally at a dose of 2 mg/g body weight. Blood samples were collected at 15, 30, 45, 60, and 120 min post-administration.

### 2.5. Homogenization of Pancreatic Tissue

Following surgical removal of the pancreatic tissue, the pancreases were weighed and stored at −80 °C until analysis. The pancreatic tissue was homogenized in 800 μL of extraction buffer (Tris base 50 mM, pH 7.4; Ethylenediamine tetraacetic acid (EDTA) 1 mM; sodium orthovanadate 2 mM; and PMSF 2 mM). After the homogenization, the samples were subjected to centrifugation, and the supernatants were analyzed for protein content.

### 2.6. Protein Quantification

The protein concentration of the pancreatic homogenate was determined using the method of Bradford et al. (1976) [11]. This method is based on determining the concentration of peptide bonds by measuring the absorbance of the protein–dye complex. The complex absorbs at a wavelength of 595 nm, with bovine serum albumin (BSA) solution at 2 mg/mL used as the standard.

### 2.7. Lipid Peroxidation Levels

The Buege and Aust (1978) colorimetric technique was employed to quantify thiobarbituric acid-reactive substances (TBARS) [12]. The samples, containing 0.3 mg of protein, were sequentially combined with 30% (*w*/*v*) trichloroacetic acid (TCA) and 10 mM TRIS buffer (pH 7.4). The mixture was then subjected to centrifugation at 1180× *g* for 10 min, and the supernatant was boiled at 100 °C for 15 min with 0.73% (*w*/*v*) thiobarbituric acid. The concentration of the pink pigment was determined by measuring the absorbance at 535 nm using a Biochrom Libra S12 visible spectrophotometer (Biochrom, Holliston, MA, USA) at room temperature, with the results expressed as mM/mg protein.

### 2.8. Assessment of Protein Oxidation Levels (Carbonyls)

The evaluation of protein oxidation was conducted using the technique described by Zanatta et al. (2013) [13]. The samples were placed in a 0.3 mg protein solution on ice. A 30% (p/v) TCA solution was added to the sample, which was then centrifuged for 14 min at 1180× *g*. The sediment was resuspended in 10 mm^2^. Then, 4-Nitrobenzhydrazine was immediately incubated in a dark room for one hour and then centrifuged three times in a solution of ethyl acetate. The sediment was resuspended in 6M of guanidine chloride, and the absorbance was read at 370 nm (37 °C) using a visible spectrophotometer, Biochrom Libra S12 (Biochrom, Holliston, MA, USA). The results are expressed as μM/mg of protein.

### 2.9. Superoxide Dismutase (SOD) Activity

A homogenate comprising 0.1 mg of protein was added to 0.05 M of carbonate buffer, which contained 0.1 mM of EDTA (pH 10.2). The action was initiated with 150 mM of epinephrine, and the SOD activity was determined by measuring the inhibition of adrenaline auto-oxidation at 30 °C. The decrease in absorbance was monitored for 1.5 min at 480 nm using a spectrophotometer, and the results were expressed as units per milligram of protein. The unit of SOD was defined as the quantity of protein necessary to inhibit the autooxidation of 1 μmol of epinephrine per minute. The SOD activity was conducted in accordance with the protocol originally established by Misra and Fridovich (1972) [14].

### 2.10. Catalase (CAT) Activity

The CAT activity was conducted in accordance with the methodology previously described by Aebi (1984) [15]. The method is based on the determination of the decomposition constant of H_2_O_2_ in our specified temperature and pH conditions, which yielded a value of 4.6 × 10^7^. Subsequently, 0.3 M of H_2_O_2_ was added to the sample (0.1 mg of protein), which was followed by the addition of phosphate buffer (50 mM, pH 7.0) at 20 °C. The decay of the absorption was then monitored for a period of four minutes at 240 nm in a spectrophotometer (Biochrom Libra S12 Visible, Holliston, MA, USA). The results are expressed as U/mg of protein. The unit of catalase was defined as the quantity of protein necessary to convert 1 μmol of hydrogen peroxide into water per minute.

### 2.11. Glutathione-S-Transferase (GST) Activity

A 0.15 mg protein sample was added to a 1 mL quartz cuvette containing potassium phosphate buffer (0.1 M), EDTA (1 mM), GSH (1 mM), and CDNB (1 mM). The absorbance at 340 nm was recorded for a period of approximately three minutes with temperature control (30 °C) in a spectrophotometer (Biochrom Libra S12 Visible, Holliston, MA, USA). The results were expressed as U/mg of protein. The term “unit of GST enzymatic activity” was defined as the quantity of the enzyme required to catalyze the formation of 1 μmol of the DNP-SG compound to minute [16].

### 2.12. Reduced Glutathione (GSH), Oxidized Glutathione (GSSG) Levels, and Cellular REDOX State (GSH/GSSG Ratio)

To assess the glutathione (GSH) levels, the samples (0.1 mg of protein) were initially diluted in 0.1 M of phosphate buffer containing 5 mM of EDTA (pH 8.0). Subsequently, an aliquot of the diluted sample was incubated with o-phthaldialdehyde at room temperature for precisely 15 min. Fluorescence intensities were quantified at 420 nm and referenced at 350 nm on a spectrofluorometer (FLUOstar Omega—BMG Labtech, Fullerton, CA, USA), and these were then compared with a standard curve of known GSH concentrations (0.25–10 nM), which were also incubated with OPT. The results of the experiment are presented in the following section. To ascertain the levels of GSSG, the samples (0.1 mg of protein) were incubated with 0.04 M of N-ethylmaleimide for 30 min at room temperature, after which 0.1 M of NaOH was added. GSH was employed to ascertain the GSSG levels. The REDOX state was determined by calculating the GSH/GSSG ratio in accordance with the methodology established by Hissin and Hilf (1976) [17].

### 2.13. Total Thiol Content (Sulfhydryl’s -SH Dosage)

The sulfhydryl content was determined through a reaction with the compound 5,5′-dithiobis (2-nitrobenzoic acid) (DTNB). An aliquot of the homogenate (0.3 mg μg of protein) was incubated in the dark with 30 μL of 10 mM DTNB, and this made up to a final volume of 1 mL with extraction buffer, as described by Habig et al. (1974) [16]. Absorbance was read on a spectrophotometer (LIBRA S12 UV/VISIBLE, Holliston, MA, USA) at 412 nm. Results were expressed as sulfhydryls (μmol/mg protein).

### 2.14. RNA Extraction and RT-PCR

RT-PCR was used to assess the genes associated with antioxidant enzymes (SOD, CAT, and GPx) and inflammatory cytokines (tumor necrosis factor alpha (TNF-α) and interleukin-6 (IL-1)). The designed primers are found in Table 2. The extraction was performed with pancreatic homogenate, which was lysed in 200 µL of a solution containing trizol and chloroform. Then, the samples were centrifuged, and the aqueous phase was transferred to another tube containing isopropanol. The resulting RNA was then washed with ethanol (75%) and centrifuged at 7000× *g* for 5 min. The RNA pellet was dried at room temperature, resuspended in RNAse-free water, and then stored at −80 °C. RNA quantification was performed by spectrophotometry (260/280 nm). Reactions for each primer group and all the parameters were evaluated using constant concentrations of RNA and according to the standards provided by the manufacturer of the SuperScript^®^ III Platinum^®^ SYBR^®^ Green One-Step qRTPCR Kit (Invitrogen, Foster City, CA, USA). In addition, the expression of the β2-microglobulin (β2M) gene was used as the normalizing gene for each sample, and the expression was quantified according to the 2^−∆∆CT^ calculation [18].

### 2.15. Histology of the Pancreas

The excised pancreases were preserved in 10% buffered formalin. They were then sectioned, and the fragments were subjected to routine histology and embedded in paraffin. From these blocks, 4 mm thick histologic sections were cut on a microtome. The sections were mounted on slides and subjected to various staining techniques, such as Hematoxylin-eosin (HE), for observation of the general histologic features [19]. The islets of Langerhans were evaluated for number of cells, cell length, islet diameter, and number of islets. The analyses were performed in ImageJ v.2.0. The exocrine and endocrine regions were analyzed by selecting two quadrants in each image. The quadrants were added by the software with an area of 1500 µm. Morphometric analyses were carried out on 1000 cells per experimental group.

### 2.16. Statistical Analysis

Data are presented as the mean and standard error of the mean. The unpaired Student’s t-test was used to compare groups at 30 days of age when only considering the diet factor. The level of significance was considered when *p* ≤ 0.05. GRAPHPAD PRISM software version 8.0 was used to generate graphs and process the statistical data.

## 3. Results

### 3.1. Murinometric Profile and Weight of the Pancreas of the Offspring at 30 Days of Life

**Figure 1:**
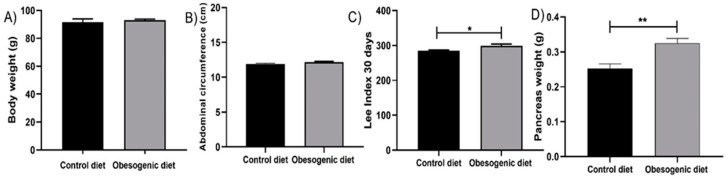
The murinometric profile and tissue weight of the pancreas of the offspring at 30 days of life. (**A**) Body weight (CD: n = 7 OD: n = 7); (**B**) Abdominal circumference (CD: n = 7 OD: n = 8); (**C**) Lee Index (CD: n = 7 OD: n = 8) (* *p* = 0.034); and (**D**) pancreas weight (CD: n = 7 OD: n = 8) (** *p* = 0.002). Data are expressed as the mean ± SEM using the unpaired Student *t* test.

After 30 days of life, the offspring were subjected to anthropometric measurements. Body weight and abdominal circumference were not statistically different. The Lee Index showed an increase in the obesogenic diet group (CD = 284.7 ± 7.026, n = 7 vs. OD: 299.0 ± 14.54, n = 8, *p* = 0.034 in grams), as seen in Figure 1C. In addition, the weight of pancreatic tissue increased in the OD group, indicating that the maternal consumption of obesogenic diet affects the weight of pancreatic tissue (CD = 0.2533 ± 0.0329, n = 7 vs. OD: 0.3249 ± 0.03926, n = 8, *p* = 0.002 in grams), as seen in Figure 1D.

### 3.2. Serum Biochemical Profile

**Figure 2:**
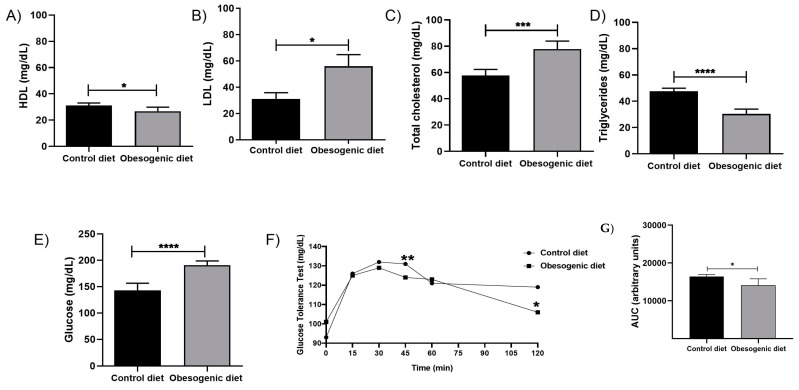
The biochemical profiles and GTT of the offspring at 30 days of life. (**A**) HDL levels, (CD: n = 6 OD: n = 7) (* *p* = 0.011); (**B**) LDL levels, (CD: n = 7 OD: n = 7) (* *p* = 0.032); (**C**) total cholesterol levels (CD: n = 5 OD: n = 5) (*** *p* = 0.004); (**D**) triglyceride levels (CD: n = 5 OD: n = 5) (**** *p* < 0.0001); (**E**) glucose levels (CD: n = 5 OD: n = 6) (**** *p* < 0.0001); (**F**) glucose tolerance test (GTT) (CD: n = 5 OD: n = 6) (** *p* = 0.007, * *p* = 0.039); and (**G**) area under the curve (AUC) of the GTT (CD: n = 5 OD = n = 6) (* *p* = 0.030). Data are expressed as the mean ± SEM using the unpaired Student *t* test.

Regarding the serological analysis, which was used to identify the serum biochemical profile of the offspring and is expressed in mg/dL, it was found that the HDL (CD:31.21 ± 0.735, n = 6 vs. OD: 26.83 ± 1.116, n = 7, *p* = 0.011) and triglyceride levels (CD: 47.70 ± 1.006, n = 5 vs. OD: 30.36 ± 1.646, n = 5, *p* < 0.0001) were decreased in the OD group (Figure 2A,D), while LDL (CD: 31.08 ± 4.823, n = 7 vs. OD: 55.99 ± 8.75, n = 7), glucose (CD: 142.9 ± 6.083, n = 5 vs. OD: 190.6 ± 3.27, n = 6, *p* < 0.0001), and total cholesterol (CD: 57.76 ± 2.052, n = 5 vs. OD: 77.80 ± 2.71, n = 5, *p* = 0.004) increased in the animals receiving the obesogenic diet (Figure 2B,C,E).

### 3.3. Oral Glucose Tolerance Test (OGTT)

To determine whether the maternal diet affected serum glucose absorption and might predispose the animals to some imbalance in glucose homeostasis, the OGTT was performed. During the first 30 min, there were no significant differences between the groups, but, at 45 min (CD: 155 ± 5.010, n = 5 vs. OD: 121.2 ± 7.846, n = 6, *p* = 0.003) and at 120 min (CD: 120.2 ± 3.089, n = 5 vs. OD: 105.5 ± 4.91, n = 6, *p* = 0.039), there was a decrease in the glucose levels in the obese group, especially in the last period (Figure 2F). The results are expressed in mg/dL.

### 3.4. Lipid Peroxidation, Protein Oxidation, and Total Thiols

**Figure 3:**
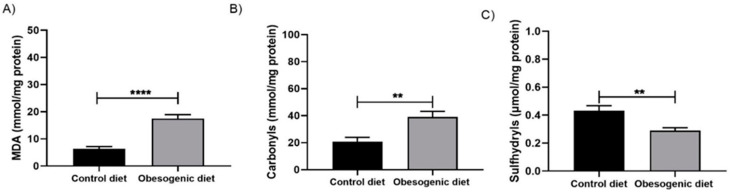
Evaluation of the lipid peroxidation, protein oxidation, and total thiols levels of the pancreatic tissue of offspring at 30 days of life. (**A**) Malondialdehyde (MDA) levels (CD: n = 6 OD: n = 6) (**** *p* < 0.0001; (**B**) carbonyl levels (CD: n = 6 OD: n = 6) (** *p* = 0.006); and (**C**) sulfhydryl levels (CD: n = 7 OD: n = 7) (** *p* = 0.005). Data are expressed as the mean ± SEM using the unpaired Student *t* test.

After obtaining the pancreatic tissue homogenate and protein dosage, assays were performed to measure oxidative stress biomarkers and total thiols. Regarding lipid peroxidation, an increase in the MDA levels was observed in the OD group (Figure 3A) (C: 6.320 ± 0.854, n = 6 vs. OD: 17.49 ± 1.448, n = 6, *p* < 0.0001), as well as an increase in the protein oxidation levels (C: 20.80 ± 3.199, n = 6 vs. OD: 39.07 ± 4.168, n = 6, *p* = 0.006) (Figure 3B). These results are expressed in μg/mg protein. Furthermore, there was also a decrease in the pancreatic sulfhydryl levels (C: 0.43 ± 0.036, n = 7 vs. OD: 0.29 ± 0.019, n = 7, *p* = 0.005). These results are expressed in M/mg protein, as seen in Figure 3C.

### 3.5. Activity of the Enzymatic Antioxidant System

**Figure 4:**
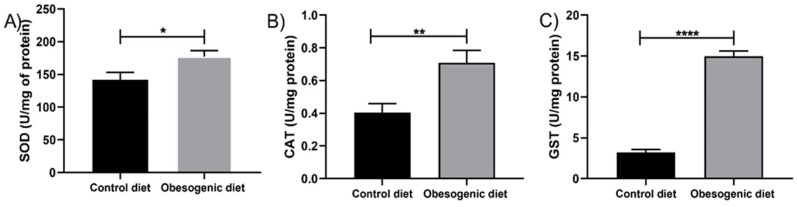
Activity of the pancreatic enzymatic antioxidant system of the offspring at 30 days of life. (**A**) Superoxide dismutase (SOD) enzymatic activity (CD: n = 6 OD: n = 7) (* *p* = 0.030); (**B**) catalase (CAT) enzymatic activity (CD: n = 7 OD: n = 6) (** *p* = 0.007); and (**C**) Glutathione-S-Transferase (GST) enzymatic activity (CD: n = 6 OD: n = 6) (**** *p* < 0.0001). Data are expressed as the mean ± SEM using the unpaired Student *t* test.

The enzymatic antioxidant system consists of a series of enzymes that are aimed at eliminating reactive species or transforming them into molecules that are less harmful to the body. The enzymatic antioxidant system was increased in the OD group in the kinetics of all the enzymes analyzed. Thus, an increase in SOD activity was observed (CD: 141.9 ± 11.61, n = 6 vs. OD: 177.4 ± 8.81, n = 7, *p* = 0.030), as well as in CAT (CD: 0.403 ± 0.05, n = 7 vs. OD: 0.707 ± 0.07, n = 6, *p* = 0.007) and GST (CD: 3.23 ± 0.36, n = 6 vs. OD:14.97 ± 0.65, n = 6, *p* < 0.0001), (Figure 4A–C). These results are expressed in U/mg of protein.

### 3.6. Activity of the Non-Enzymatic Antioxidant System

**Figure 5:**
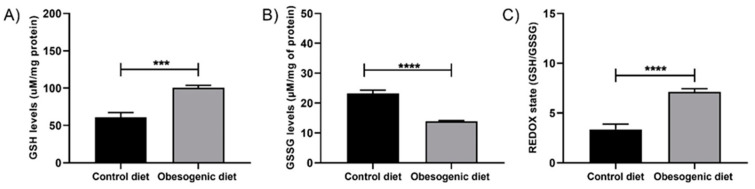
The non-enzymatic antioxidant systems of the offspring at 30 days of life. (**A**) GSH levels (CD: n = 7 OD: n = 7), (*** *p* = 0.0001); (**B**) GSSG levels (CD: n = 6 OD: n = 7) (**** *p* < 0.0001); and (**C**) REDOX state (CD: n = 6 OD: n = 7) (**** *p* < 0.0001). Data are expressed as the mean ± SEM using the unpaired Student *t* test.

The activity of the non-enzymatic antioxidant system was evaluated by the GSH and GSSG contents in the pancreatic homogenate and then by performing the REDOX balance test, which was conducted in correspondence with the GSH/GSSG ratio. At 30 days of age, the GSH levels (Figure 5A) were increased in the OD group (CD: 60.85 ± 6.51, n = 7 vs. OD: 100.8 ± 3.15, n = 7, *p* = 0.0001), while GSSG (Figure 5B) was decreased in the OD group (CD: 23.28 ± 1.08, n = 6 vs. OD: 13.89 ± 0.27, n = 7, *p* < 0.0001). Regarding the REDOX state (Figure 5C), an increase was observed in the OD group (CD: 3.36 ± 0.51, n = 6 vs. OD: 7.12 ± 0.31, n = 7, *p* < 0.0001).

### 3.7. Analysis of the Gene Expression of Antioxidant Enzymes and Inflammatory Cytokines in the Pancreatic Tissue of the Offspring

**Figure 6:**
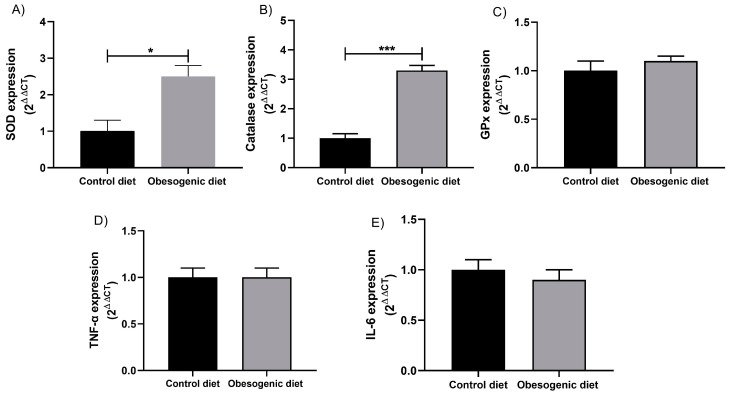
Gene expressions of the antioxidant enzymes and inflammatory factors of the pancreatic tissue of the offspring at 30 days of life. (**A**) SOD expression (CD: n = 4 OD: n = 3) (* *p* = 0.018); (**B**) catalase expression (CD: n = 4 OD: n = 3) (*** *p* = 0.0002); (**C**) GPx expression (CD: n = 4 OD: n = 4); (**D**) TNF-α expression (CD: n = 4 OD: n = 4); and (**E**) IL-6 expression (CD: n = 4 OD: n = 4). Data are expressed as the mean ± SEM using the unpaired Student *t* test.

Regarding the gene expression of the antioxidant enzymes, it was seen that SOD (Figure 6A) increased (CD: 1 ± 0.30, n = 4 vs. OD: 2.50 ± 0.30, n = 3, *p* = 0.018), and the same also occurred with CAT (CD: 1 ± 0.150, n = 4 vs. OD: 3.30 ± 0.173, n = 3, *p* = 0.0002) (Figure 6B), although GPx (Figure 6C) did not show a significant difference. The evaluation of inflammatory process was analyzed by IL-6 (Figure 6D) and TNF-α expression (Figure 6E), and these results are expressed and were quantified according to the 2^−∆∆CT^ calculation. The results showed no significant difference in any of the inflammatory markers evaluated.

### 3.8. Histology and Histomorphometry Analysis of the Pancreas

**Figure 7:**
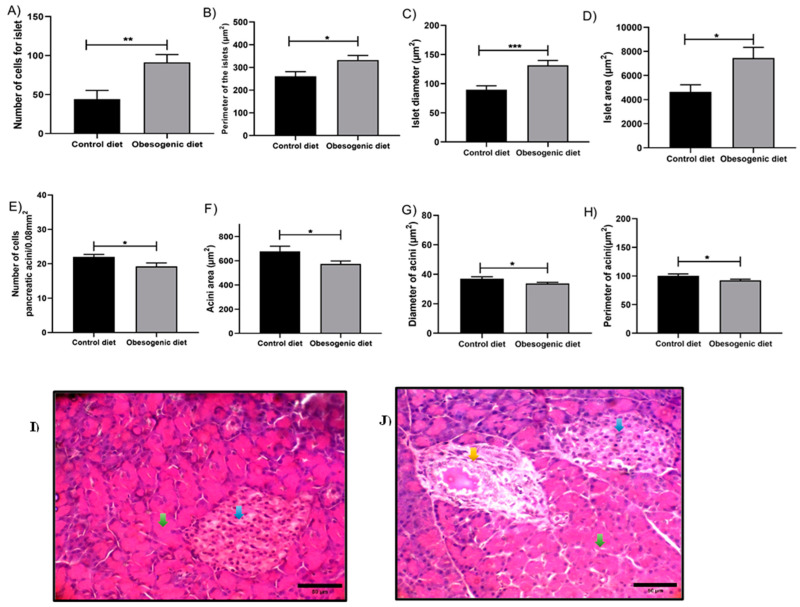
The histomorphometries and histologies of the pancreatic tissue of the offspring at 30 days of life. (**A**) Number of cells for islet (CD: n = 6, OD: n = 6) (** *p* = 0.007); (**B**) perimeter of the islet (CD: n = 6, OD: n = 6) (* *p* = 0.015); (**C**) islet diameter (CD: n = 6, OD: n = 6) (*** *p* = 0.0005); (**D**) islet area (CD: n = 6, OD: n = 6) (* *p* = 0.011); (**E**) number of cells in the pancreatic acini (CD: n = 6, OD: n = 6) (* *p* = 0.031); (**F**) acini area (CD: n = 6 OD: n = 6) (* *p* = 0.022); (**G**) diameter of acini (CD: n = 6 OD: n = 6) (* *p* = 0.024); (**H**) perimeter of acini (CD: n = 6 OD: n = 6) (* *p* = 0.028); (**I**) photomicrograph of the pancreas of the control animals; (**J**) photomicrograph of the pancreas of the obesogenic animals. Blue arrows indicate pancreatic islets. Green arrows indicate pancreatic acini. The yellow arrow indicates a damaged islet with vacuoles in its center. Data are expressed as the mean ± SEM using the unpaired Student’s *t* test.

Histomorphometry analysis of the exocrine portion of the tissue, corresponding to the pancreatic acini, revealed a reduction in cell number in the OD group (CD:22 ± 0.72, n = 6 vs. OD: 19.23 ± 1,00, n = 6, *p* = 0.031) (Figure 7A). In addition, there were reductions in the area that was expressed in the acini area (µm^2^) (C: 678.7 ± 41.73 vs. OD: 573.3 n = 6, *p* = 0.022) (Figure 7D), the diameter that was expressed in the diameter of the acini (µm^2^) (CD: 37.04 ± 1.24, n = 6 vs. OD: 33.76 ± 0.83, n = 6, *p* = 0.024) (Figure 7C), and the perimeter of the acini that was expressed in the perimeter of the acini (µm^2^) (CD: 100 ± 3.185, n = 6 vs. OD: 92.25 ± 2.12, n = 6, *p* = 0.028) (Figure 7B). In contrast, the endocrine portion associated with the pancreatic islets demonstrated an increase in all analyzed parameters, including the number of cells (CD: 44.29 ± 11.08, n = 6 vs. OD: 91.36 ± 10.01, n = 6, *p* = 0.007) (Figure 7E); the area that was expressed in islet area (µm^2^) (CD: 4650 ± 589.2, n = 6 vs. OD: 7454 ± 887.4, n = 6, *p* = 0.011) (Figure 7F); the perimeter that was expressed in perimeter of the islets (µm^2^) (CD: 261 ± 20.66, n = 6 vs. OD: 332.7 ± 19.81, n = 6, *p* = 0.015) (Figure 7H); and the diameter that was expressed in islet diameter (µm^2^) (CD: 89.79 ± 6.65, n = 6 vs. OD: 131.4 ± 8.59, n = 6, *p* = 0.005) (Figure 7G) in the OD group. Furthermore, the presence of damaged islets and an increase in vacuoles in their center with the presence of red blood cell infiltrating inside them was identified in the OD group. However, there were no evident lymphocytic infiltrates in the pancreatic tissue, as can be seen in Figure 7I,J.

## 4. Discussion

The objective of our study was to ascertain the effects that a maternal diet with high saturated fat content, when it is offered during critical periods of the development, has on the pancreatic oxidative balance of offspring, as well as its influence on the morphological aspects of the islets and metabolic parameters associated with glucose metabolism and the serum biochemical profile of the animals.

The results of the murinometric profiles indicate that the maternal diet causes increases in the Lee Index and in the weight of the pancreas in the offspring at 30 days of life, but the body weight was not found to be statistically different. These findings indicate an increased propensity for obesity with augmented pancreatic tissue. A study conducted by [20] utilizing a maternal obesogenic diet model revealed that male and female offspring at 21 days of life exhibited no alterations in their body weight and lean mass, a finding that is consistent with our data. However, the same study demonstrated that the total body fat ratio was elevated in the group that was subjected to the dietary regimen [20]. Although it is already established that the consumption of an obesogenic diet can contribute to the development of obesity, as evidenced by the findings of [21] with Sprague Dawley rats, it was demonstrated that the consumption of high-fat diet for a period of 12 weeks resulted in the development of obesity, and this was accompanied by an increase (36.9%) in body weight and elevated glycemic levels at both 45 and 120 min during the oral glucose tolerance test (OGTT) and insulin resistance test.

Furthermore, alterations in glucose tolerance were observed in our study. Initially, no differences were evident in the obese diet (OD) animals during the first 30 min. However, at 45 min and 120 min, a decline in glucose levels was noted in the OD group, particularly during the latter period. Other studies demonstrate that animals subjected to diets that promote obesity exhibit alterations in glucose homeostasis. Additionally, there was a notable elevation in the serum glucose levels, and this was accompanied by an increase in the total cholesterol, triglycerides, and low-density lipoprotein (LDL) levels, with a concomitant reduction in high-density lipoprotein (HDL). These results show that consumption of the maternal diet increases lipid and blood glucose levels, indicating a possible dysregulation in the biochemical profile of the animals, which may be related to changes in pancreatic and hepatic metabolism. Furthermore, in the hepatic tissue, there is a discernible rise in cholesterol and triglyceride levels [3,21]. The dysregulation of cholesterol and triglycerides may indicate the presence of dyslipidemia or may compromise the lipid metabolism, thereby contributing to tissue damage and the overall health of the animal. Moreover, the study performed by [21] demonstrated that a diet high in fructose is associated with an increase in the size of epididymal adipose tissue and serum insulin levels, which can potentially result in glucose homeostasis complications.

Modifications in glucose homeostasis and lipid metabolism have the potential to impact the pancreas, resulting in increases on the hormone production and release. Additionally, these changes can contribute to increases in oxidative stress on the gland. Our findings indicate that, with respect to oxidative parameters, there was elevation in the malondialdehyde (MDA) and carbonyls levels in the OD group. Additionally, there was an increase in the activity of the superoxide dismutase (SOD), catalase (CAT), and glutathione-S-transferase (GST) enzymes, as well as an increase in reduced glutathione (GSH) levels, while the oxidized glutathione (GSSG) levels decreased, and the REDOX balance enhanced in the obesogenic group. A systematic review study carried out by [22] demonstrated that increases in islet mass resulting from a minor inflammatory process can disrupt the oxidative balance and elevate production of reactive oxygen species (ROS). This cascade of dysregulation impacts the biogenesis and self-checking mechanisms of beta cells, contributing to the initial stages of type 2 diabetes development. In concordance with this viewpoint, the study conducted by [22] demonstrated that prolonged dietary overnutrition elevates glucose and free fatty acids (FFA) levels in the blood. This impairs the functionality of beta cells due to lipotoxicity through various types of G protein-coupled receptors (GPRs), which may be associated with the genesis of oxidative imbalance.

Moreover, the literature indicates that increases in the SOD activity and GSH levels are associated with elevation in lipid peroxidation and protein oxidation, as well as a decline in antioxidant activity. This phenomenon can potentially contribute to the oxidative stress in animals that are fed a high-fat diet and/or an obesogenic diet [3,4]. In a study employing an obesogenic diet model in C57/BL6J mice, increases in the gene expressions of Gpx1, NRF2, and Ppargc1a were identified, suggesting an attempt by the animals to combat ROS in response to the nutritional insult [8]. In another study, conducted by [23], animals were fed with an obesogenic diet for a period of 18 months. It was found that the obese animals demonstrated significant elevation in plasma SOD activity. This phenomenon may have been associated with the increased levels of glucose and free fatty acids (FFA) present in their bloodstream. Although these studies evaluate the effects of the reactive species in animals subjected directly to the diet, our results with the offspring of obese mothers present similar changes, indicating that the consumption of a maternal obesogenic diet can compromise the oxidative metabolism, thereby increasing the reactive species production and enzymatic activity to combat the ROS excess. Furthermore, the increase in FFA and plasma glucose may act as an insult to induce this process. These results must be associated with the body’s attempt to combat the excess of reactive species produced since the biomarkers (MDA and carbonyls levels) are elevated. The association of the results of biomarkers with enzymatic activity shows that, even with increased antioxidant defenses, the pancreatic tissue is still damaged at 30 days of age.

In addition to affecting the activity of the antioxidant system enzymes, maternal nutritional insult could increase the expression of SOD and CAT. However, no increase in the expression of genes associated with the inflammatory profile, nor for GPx, was identified in our study. It should be noted that recent studies have not directly evaluated the changes in the gene expression of pancreatic enzymes in obese animals. The literature has already documented changes in the gene expression of antioxidant enzymes and genes associated with mitochondrial biogenesis. In the obese animal’s group, there was an increase in the expression of p53 associated with DNA damage but a decrease in the expression of GPx1. Additionally, there was an increase in the expression of the NFR2 and receptor activated by peroxisome proliferators gamma (PPARγ) genes associated with mitochondrial biogenesis [8]. Recent studies have demonstrated that the consumption of a high-fat and/or obesogenic diet activates the mitochondrial biogenesis through NFR2. This evidence supports the assertion that the expression of NRF2 is an essential transcription factor for maintaining neonatal REDOX balance, biogenesis, mitochondrial function, and beta cell growth. Furthermore, it has also been implicated in the preservation of functional beta cell mass in adulthood under metabolic stress [23,24].

Following the identification of the maternal nutritional insult as the cause of several pancreatic and biochemical changes, we propose an evaluation of potential structural changes in the pancreatic tissue. The results demonstrate that the animals of the OD group exhibited morphological alterations in the pancreatic tissue, including increases in the number of cells, area, diameter, and perimeter of the islets. It has been established in the literature that nutritional insults are associated with the development of tissue hypertrophy and the infiltration of inflammatory cells. Moreover, the number and the diameter of islets were increased in animals fed a diet high in refined carbohydrates [24].

As demonstrated in the experimental and clinical study conducted by [25], there is significant correlation between obesity and type 2 diabetes (DM2) in humans. Individuals with pancreatic DM2 exhibited elevated rates of angiopathy and greater total number of lymphocytes T, yet no discernible difference was found in the number of adipocytes. Although total pancreatic fibrosis was increased in both type 1 diabetes (DM1) and DM2, the patterns were distinct, with lobular and parenchymal fibrosis occurring more frequently in DM2. The rise in fibrotic processes associated with islet hyperplasia has the potential to impair the functionality of the gland. As evidenced in our study, morphological changes in islets can also lead to alterations in the oxidative balance and serum plasma glucose levels in the offspring.

Our findings demonstrate that a maternal diet high in saturated fats and sugars during critical periods of development (gestation and pregnancy) can influence the metabolic profile of the offspring at 30 days of age. This is evidenced by alterations in the pancreatic tissue weight and Lee Index, as well as the changes in the biochemical profile, which suggest potential dysfunctions in glucose homeostasis and lipid metabolism. These changes, at least in part, may have a genesis associated with changes in the pancreatic REDOX balance, including oxidative stress biomarkers and modulation of the antioxidant defenses, as well as in morphological modifications in the pancreatic islets and in the exocrine portion of the gland. The aggregation of these alterations may evoke a predisposition to DM2 or non-alcoholic pancreatic fatty disease. Therefore, a nutritional maternal environment during critical periods of development is fundamental for the maintenance of offspring health.

## 5. Conclusions

The maternal obesogenic diet has been observed to alter offspring metabolic processes as early as 30 days post-natal, with evidence of dysregulation in glucose homeostasis and plasma lipid levels. Moreover, it induces oxidative stress in pancreatic tissue, which is accompanied by morphological changes and alterations in the gene expression of the pancreatic antioxidant enzymes. This may predispose the offspring to the development of pathologies, such as type 2 diabetes mellitus (DM2). However, further studies are required to elucidate the entire mechanism involved in these changes and to what extent they can compromise the health of the offspring.

## Figures and Tables

**Table 1:** Composition of the ingredients utilized in the formulation of the experimental diets.

Ingredient in g per 100 g of Diet	Presence (g)	High-Fat Diet (g)
Maize starch	-	11.5
Wheat flour	-	12.0
Maisena cookie	-	7.2
Soy flour	-	8.5
Pig lard	-	5.5
Milk cream	-	3.0
Margarina (65% lipids)	-	3.5
Casein (>85%)	-	20.0
Sucrose	-	20.0
Soy oil	-	4.0
Fiber (cellulose)	-	0.3
Mineral mix	-	2.5
DL-methionine	-	0.3
Choline bitartrate	-	0.25
BTH	-	0.0014
Sodium chloride	-	0.036
Total (g)	-	100.0
Kcal/100 g	3.44	4.42
% Total fat	11.0	31.5
% Protein	28.0	19.6
% Carbohydrates	61.0	49.3

Source: The Presence^®^ vivarium diet is a high-fat diet based on the study by Ferro Cavalcante et al. (2013) [10]. The Mineral Mix is composed of the following reagents (in mg/kg of diet): CaHPO_4_, 17.200; KCl, 4000; NaCl, 4000; MgO, 420; MgSO_4_, 2000; Fe_2_O_2_, 120; and FeSO_4_·7H_2_O, 200 (Ferro Cavalcante et al., 2013, [10]).

**Table 2:** List of the primers used for PCR.

Primer	Forward	Reverse
β2M	TGA CCG TGA TCT TTC TGG TG	ACT TGA ATT TGG GGA GTT TTC TG
GPx	ATC AGT TCG GAC ATC AGG AG	CCT CGC ACT TCT CAA ACA AT
SOD	GCG ACC TAC GTG AAC CT	CAG CAA CTC TCC TTT GGG TT
CAT	AGA AAC CCA ACT CAC CT	TGA GCC ATA GCC ATT CAT GT
TNF-α	AAG CAT GAT CCG AGA TGT GG	AGT AGA CAG AAG AGC GTG GT
IL-1	GCG GTT CAA GGC AT ACA GC	GCC ATA GCT TCA GAC AC

## Data Availability

The data generated or analyzed during this study are provided in full in the published article. If requested by the journal, the gross values may be sent.

## Abbreviations

The following abbreviations are used in this manuscript:

DTNB	5,5′-dithiobis (2-nitrobenzoic acid)
IBGE	Brazilian Geography and Statistics
CAT	Catalase
CD	Control diet group
CEUA	Ethics Committee for the Use of Animals
FFA	Free fatty acids
GLUT	Glucose transporters
GPRs	G protein-coupled receptors
GPx	Glutathione peroxidase
GST	Glutathione-S-Transferase
HE	Hematoxylin-Eosin
HDL	High-density lipoprotein
SOD	Superoxide dismutase
IL-6	Interleukin-6
LDL	Low-density lipoprotein
MDA	Malondialdehyde
NRF1	Nuclear respiratory factor 1
NRF2	Nuclear respiratory factor 2
OD	Obesogenic diet
OGTT	Oral Glucose Tolerance Test
GSSG	Oxidized glutathione
ROS	Reactive oxygen species
PPARγ	Receptor Activated by Peroxisome Proliferators Gamma
GSH	Reduced glutathione
TBARS	Thiobarbituric acid-reactive substances
TCA	Trichloroacetic acid
TNF-α	Tumor Necrosis Factor Alpha
DM2	Type 2 diabetes
WHO	World Health Organization
